# Moving bodies, healing bonds: dyadic embodied psychotherapy in crisis settings

**DOI:** 10.3389/fpsyg.2025.1714477

**Published:** 2026-01-12

**Authors:** Maya Vulcan, Tamar Dvir, Einat Shuper Engelhard

**Affiliations:** 1 The School of Creative Art Therapies, Faculty of Social Welfare and Health Sciences, University of Haifa, Haifa, Israel; 2 The School of Creative Art Therapies, Faculty of Humanities and Social Sciences, Kibbutzim College of Education, Tel Aviv, Israel

**Keywords:** crisis, dance movement therapy, displacement, dyadic psychotherapy, embodied interventions, emergency settings, parent–child, trauma

## Abstract

**Introduction:**

Children and parents exposed to war-related forced displacement often experience disruptions in emotional regulation, relational availability, and communicative capacities. While dyadic interventions are increasingly recognized as important in emergency contexts, less is known about how movement-based, embodied processes operate within parent-child therapeutic work during acute displacement-related crises. This qualitative study examines psychotherapists' experiences of movement-based interventions with parent-child dyads in emergency settings, focusing on therapists' subjective understandings of therapeutic processes with children and their caregivers.

**Methods:**

Using Interpretative Phenomenological Analysis (IPA), semi-structured interviews were conducted with ten psychotherapists trained in movement psychotherapy and experienced in crisis and emergency contexts. Interviews explored therapists' meaning-making regarding therapeutic change within embodied dyadic work following forced displacement due to war.

**Results:**

Two superordinate themes emerged. First, the dyadic frame was perceived as critical for supporting embodied co-regulation and emotional availability in both parent and child. Therapists described how caregivers' presence, initially peripheral, often evolved into active engagement, enhancing the child's sense of safety and enabling reciprocal transformation within the dyad. Second, movement was understood not merely as a technique but as a primary therapeutic modality, facilitating preverbal expression, relational attunement, and regulatory embodied meaning-making in contexts where verbal discourse was limited. Therapists' embodied presence frequently served to model parental functions, reanimating caregiver responsiveness and vitality. Movement also functioned as a diagnostic and relational lens, allowing real-time assessment of trauma responses, interactional synchrony, and regulatory shifts.

**Discussion:**

Findings highlight the value of integrating nonverbal, developmentally sensitive, and relationally oriented approaches into clinical frameworks for families experiencing displacement-related crises. The study suggests that intervention design in emergency contexts should explicitly include dyadic and body-based components that engage both caregiver and child as active participants, emphasizing embodied co-regulation and relational repair as central processes in the early stages of trauma recovery.

## Introduction

The humanitarian crises of the past decade, including war, natural disasters and forced displacement, have exposed millions of children worldwide to severe psychological trauma. These catastrophic events not only pose immediate threats to the physical survival of the victims but may also create long-term disruptions to the development and well-being of children who survived, resulting in lasting emotional, relational, and cognitive consequences (Bürgin et al., 2022). Wisner and Adams (2002) define a *complex emergency* as a situation characterized by disrupted livelihoods and threats to life due to warfare, civil unrest, or large-scale displacement, often requiring emergency responses under politically and physically hazardous conditions. Such situations are characterized by the collapse of social infrastructures and the protective systems which normally support children's mental health, and generate profound individual and collective trauma.

The present study was conducted in the aftermath of the October 7 terror attacks in Israel, during which many families spent many hours in shelters under threat of ongoing violence. Following these events, numerous families in southern Israel were forced to leave their homes amid intense conflict in the area. Many were relocated to temporary facilities, sometimes separated from other family members (Shay et al., 2024; Weinberg, 2025). In the wake of such multifaceted harms, including those experienced after the October 7 attacks, mental health professionals rely on crisis-focused interventions designed to mitigate distress and support resilience in children and parents (Arega, 2023). A growing body of meta-analytic evidence supports the efficacy of trauma-focused interventions for children affected by humanitarian crises (Burkhart et al., 2023; Nocon et al., 2017; Velu et al., 2025), confirming the positive impact of trauma-focused approaches such as Cognitive Behavioral Therapy, Eye Movement Desensitization and Reprocessing, and Narrative Exposure Therapy in alleviating psychological distress among refugees and children exposed to disasters. Alongside the proliferation of school- and community-based psychosocial interventions for children, it is evident that delivering mental health interventions in emergency contexts presents a range of specific operational challenges, including limited infrastructure, high population mobility, and complex cultural dynamics (Kamali et al., 2020).

Two widely implemented frontline interventions in early trauma response are Play Therapy and Psychological First Aid (PFA). Play therapy provides children with a symbolic and developmentally appropriate medium for expressing overwhelming experiences (Cohen et al., 2010) and has shown efficacy in reducing symptoms of posttraumatic stress and anxiety among children exposed to war and displacement (Cohen and Gadassi, 2018; Schottelkorb et al., 2012; Tucker et al., 2021). PFA, in contrast, is a brief, low-intensity intervention designed to restore a sense of safety, stability and social connection in the immediate aftermath of crisis (Gilbert et al., 2021; Wang et al., 2024). Together, these approaches highlight two essential needs in early intervention: emotional expression and environmental stabilization. However, neither modality explicitly engages embodied, movement-based processes within the parent–child dyad, nor do they address the therapist's embodied role in crisis contexts.

### Integrating caregivers into psychosocial interventions for children in crisis

Caregivers play a pivotal role in the emotional recovery of children affected by crisis and displacement and their involvement in mental health interventions has been consistently associated with improved treatment outcomes (Szota et al., 2023). Research has demonstrated that constructive caregiver involvement in child mental health treatment is associated with better session attendance, greater adherence to treatment protocols, enhanced clinical outcomes, and increased application of therapeutic skills beyond the therapy setting (Rependa et al., 2024). However, chronic post-displacement stressors, such as economic hardship and ongoing insecurity, can erode caregivers' emotional resources, contributing to parenting strain, withdrawal, reduced warmth, and impaired parent–child interactions (Sim et al., 2018), underscoring the need for contextualized caregiver support. Meta-analytic findings provide empirical support for the inclusion of caregivers in trauma interventions, showing that youth exposed to trauma experience reductions in posttraumatic stress disorder (PTSD), depression, and anxiety symptoms when caregivers were involved (Arega, 2023; Szota et al., 2023). Despite these understandings, structured dyadic therapies, those that explicitly target the parent–child relationship as the unit of intervention, remain rare in the acute phases of emergency response. Nocon et al. (2017) note that even when parental involvement is intended, as in child-centered play therapy, actual participation is often limited. This underscores the need for more intentional and structured dyadic designs, particularly in high-stress, resource-constrained environments.

Typically, caregiver participation in emergency interventions is informal and auxiliary, limited to psychoeducation or practical guidance, rather than embedded in systematic, dual-focused therapeutic models (Kamali et al., 2020). This gap is reinforced by findings from Bauch (2022), who, in a comprehensive review of creative arts therapy literature with refugee families, identified a persistent disconnect between field-based practice and peer-reviewed research. While NGO-led interventions often involve parents or caregivers in creative ways, few studies rigorously document or evaluate the mechanisms and outcomes of these dyadic efforts. As a result, many interventions remain primarily child-focused, with limited assessment of family-level dynamics or the relational effects of caregiver participation (Arega, 2023).

### Dyadic psychotherapy in trauma: from attachment theory to creative arts integration

Parent–child psychotherapy, also known as dyadic therapy, is grounded in attachment theory and psychodynamic principles. It seeks to restore disrupted attachment systems by facilitating the co-exploration of emotional experiences between parent and child, particularly in the aftermath of trauma (Cicchetti and Toth, 2009; Fonagy et al., 2002). A leading model within this domain is Child–Parent Psychotherapy (CPP), developed by Lieberman et al. (2005). Building on the metaphor of “ghosts in the nursery,” (Fraiberg et al., 1975), they introduced the concept of “angels in the nursery”—internalized experiences of attuned care that can be reactivated to support healing. This model emphasizes that dyadic trauma therapy involves not only the processing of past harm, but also a reawakening of internal representations of safety, joy, and connection. These “angels” may be revived through the child's presence and co-regulated interaction, enabling caregivers to reconnect with their role as protective and emotionally available figures. CPP employs a range of techniques, including reflective functioning (Slade, 2005), and the interpretation of behavior through relational history (Fraiberg et al., 1975), and is grounded in verbal-symbolic interaction. CPP has demonstrated positive outcomes in trauma-exposed families, particularly in improving emotional regulation and relational security (Lieberman et al., 2005). Despite these promising models, the implementation of structured dyadic interventions in acute emergency settings remains limited. Most available programs either focus solely on the child or involve parents only in peripheral or supportive roles (Arega, 2023; Bürgin et al., 2022). Structural barriers, including displacement, instability, and the inaccessibility of private therapeutic spaces, further constrain the feasibility of delivering intensive, family-centered care in such environments.

Neuroscientific research increasingly supports the unique contribution of creative arts therapies in trauma care (Perryman et al., 2019). Malchiodi (2020) emphasizes that trauma is encoded somatically and stored in implicit memory, making body-based approaches particularly effective for activating sensory pathways, supporting co-regulation, and enabling the symbolic integration of overwhelming experiences. This aligns with trauma research emphasizing the body's role in recovery, and the need for interventions that access non-verbal, sensorimotor memory (van der Kolk, 2014). Drawing on psychodynamic theory, attachment models, sensorimotor processing, and trauma-informed care, Dance Movement Therapy (DMT), a body-oriented psychotherapeutic modality under the Creative Arts Therapies (CAT) umbrella, offers a therapeutic space for emotional regulation, relational repair, and embodied safety, particularly in contexts where verbal communication may be developmentally or culturally limited (Baumgarten et al., 2022; Crooks and Mensinga, 2021; Schaeffer and Cornelius-White, 2021). Movement-based activities with refugee families foster emotional connection and strengthen resilience through non-verbal interaction (Baumgarten et al., 2022). Dieterich-Hartwell et al. (2021) developed a structured, trauma-informed DMT protocol for resettled refugees, emphasizing culturally rooted rituals, symbolic props, and shared dance metaphors as mechanisms for identity repair, emotional regulation, and relational support. These findings underscore DMT's potential to promote recovery through embodied co-creation and community-based healing. Common therapeutic techniques across body-based interventions with refugees include the use of ritual, metaphor, and the creation of embodied “safe spaces” (Schaeffer and Cornelius-White, 2021).

While psychodynamic models like CPP emphasize verbal-symbolic and reflective processes in dyadic trauma repair, DMT offers a parallel, yet distinct, pathway grounded in non-verbal, embodied interaction. Dyadic DMT emphasizes physical synchrony, sensory attunement, and movement-based co-regulation between caregiver and child. Dyadic movement sessions enhance emotional attunement and relational closeness among parents and their children, particularly through rhythmic synchrony and shared embodied presence (Kedem and Regev, 2021). Research highlights several key mechanisms through which DMT supports dyadic healing, including embodied co-regulation that restores attachment bonds through joint rhythmic movement (Baumgarten et al., 2022), the use of non-verbal, intergenerational rituals such as rocking and shared movement for emotional expression and containment (Schaeffer and Cornelius-White, 2021), and movement-based techniques that foster self-regulation and resilience in children (Harris, 2007). Together, these mechanisms illustrate how DMT facilitates both physiological attunement and symbolic emotional processing within the caregiver-child relationship.

### The current study

Despite strong theoretical and clinical foundations, research on dyadic DMT in acute emergency settings remains limited. Existing studies have primarily examined movement-based or creative arts interventions in post-acute or resettlement phases (Dieterich-Hartwell et al., 2020), leaving a gap in understanding how therapists use movement within parent–child dyads during the initial, high-stress stages of crisis. Little is known about how therapists experience this work, how they interpret movement-based processes under emergency conditions, or how these interventions support regulation, connection, and meaning-making when families are displaced and emotionally overwhelmed. Hence, the purpose of the present study was to explore psychotherapists' lived experiences of delivering dyadic, movement-based interventions with parents and children during the acute phase of displacement following the October 7 attacks. The study investigated the following research questions:

1. How do psychotherapists experience and interpret their use of dyadic movement-based therapy with parent–child pairs in emergency settings following displacement?
2. What therapeutic dynamics emerge within the parent–child dyad in these contexts, and how are benefits and challenges of dyadic work perceived?

## Methods

### Study approach

This study employed Interpretative Phenomenological Analysis (IPA) as its qualitative approach, chosen for its emphasis on how participants make sense of their lived experiences (Smith et al., 2009). IPA is well suited to exploring complex and subjective phenomena, supporting the identification of shared experiential patterns while preserving the uniqueness of individual accounts. A central feature of IPA is its double hermeneutic, in which researchers seek to understand how participants interpret their own experiences (Montague et al., 2020). This interpretive stance aligned with the aims of the present study, enabling a nuanced exploration of how psychotherapists reflect on and construct meaning around their use of movement-based dyadic interventions in emergency contexts. By foregrounding therapists' lived experience, IPA provided a method for capturing the layered, evolving understandings that emerged within parent–child sessions conducted in emergency care settings.

### Participants

Ten psychotherapists were recruited for this study. Their clinical experience ranged from 7 to 36 years (M = 16), and all but one had extensive backgrounds in dyadic therapy, with 5 to 24 years of experience (M = 10). Participants were selected based on the following inclusion criteria: (1) direct involvement in emergency interventions during periods of collective trauma; (2) clinical experience working with both children and parents; (3) provision of treatment to at least 10 parent-child dyads during the emergency period; and (4) formal training in both psychotherapy and movement-based therapeutic modalities. All participants held a postgraduate degree in DMT from academic institutions accredited by the Israel Council for Higher Education.

#### Context of the participants' clinical work

All the participants worked with families displaced from southern Israel following the October 7 attacks and the subsequent evacuation due to ongoing conflict. These interventions began within a few days to few weeks after the attacks and continued throughout the initial months of displacement. The dyadic sessions took place in a range of emergency settings including hotels for evacuees, community resilience centers, and municipal shelters, often in improvised or multi-use rooms during the acute phase of displacement, when families were still living with uncertainty, intermittent alarms, and rapidly changing conditions. During this period, therapists worked with numerous families, including dozens of children aged 3 to 11 and their parents, all of whom had been uprooted due to active combat or the destruction of their homes. While most of the children had not directly witnessed violence, they had endured prolonged stays in shelters and were exposed to significant parental anxiety and emotional distress. No separate parental supervision sessions were conducted; instead, parental reflection and support were integrated into the dyadic work itself as part of the shared therapeutic process.

### Procedure

Participant were recruited through professional networks and clinical associations and the study employed purposive sampling (Smith et al., 2009), a method appropriate for identifying participants with the capacity to provide rich, experience-based insights into the research topic. Following initial contact, therapists who expressed interest in the study received detailed written information regarding its aims, procedures, and ethical safeguards. Those who agreed to participate signed an informed-consent form. All interviews were conducted via Zoom, according to the participants' availability and preference. Each interview lasted between 60 and 90 min and was audio-recorded. At the time of data collection, all therapeutic work relevant to this study had already been completed, ensuring that interviews focused on reflective accounts rather than ongoing clinical processes.

This study received approval from the Institutional Review Board (IRB) of the Faculty of Social Welfare and Health Sciences at the University of Haifa (Approval No. 132/24). Participants were informed of their rights, including the right to withdraw at any point without penalty. Strict confidentiality protocols were maintained throughout: all identifying details related to participants and their clients were removed, coded, and securely stored. The study adhered to established ethical standards for qualitative research involving human participants, with particular attention to the sensitive nature of trauma-related material and the clinical context in which narratives were shared.

### Data collection

Data for this qualitative study were collected using two primary instruments: semi-structured in-depth interviews (Kvale, 2007) and the Hebrew version of the Helpful Aspects of Therapy (HAT) form (Azoulay and Orkibi, 2018; Gavron et al., 2024; Llewelyn et al., 1988; Shakarov et al., 2019). The semi-structured interviews followed an IPA-consistent format, using open-ended guide questions with flexible prompts that allowed participants to elaborate on their lived experiences and meanings (Smith et al., 2009). The interview schedule explored key areas of the therapists' professional experiences and clinical reflections, including: (a) professional background and motivation for using movement-based therapy; (b) core characteristics of working with parent-child dyads in emergency contexts; (c) clinical insights and recommendations derived from working in crisis settings; (d) connections between trauma and movement, and how trauma is expressed and processed in the body; (e) perceived strengths and limitations of the intervention model; and (f) the role of movement as an observational and therapeutic tool in dyadic interactions.

The Helpful Aspects of Therapy (HAT) form (Llewelyn et al., 1988) includes two open-ended questions addressing helpful and hindering events in therapy and two rating items assessing the perceived importance of each event. The Hebrew version used in this study is an adaptation of the original form, validated for use among Israeli populations (Azoulay and Orkibi, 2018; Gavron et al., 2024; Shakarov et al., 2019). The form enables participants and practitioners to describe in their own words what they found most helpful or hindering during therapy and to rate the significance of these experiences. The HAT form complemented the interviews by identifying specific moments therapists perceived as helpful or hindering during therapy sessions, offering additional insight into the impact and efficacy of the intervention.

### Data analysis

Data were analyzed using the principles of IPA (Smith et al., 2009), to explore how psychotherapists interpreted their work delivering movement-based dyadic interventions in emergency settings. The analysis followed a structured, multi-phase process. Each interview was first transcribed verbatim and examined to identify meaningful segments within participants' narratives. A cross-case comparison was then conducted to reveal recurring patterns and shared conceptual elements, while also attending to both convergence and divergence across participants' accounts in order to preserve individual nuances and variations in emphasis. Although the analysis considered potential divergence, the data did not reveal meaningful differences that challenged the emerging themes. The highly uniform and intense emergency context appeared to elicit shared experiential themes across therapists. Emerging concepts were then grouped into categories, each labeled to reflect its core meaning. The relationships between categories were examined to develop overarching themes that captured both commonalities and subtle variations in the participants' experiences.

Selected quotations were included to illustrate key themes. To convey the prevalence of responses, the following terms were used: “*all therapists”* referred to the full sample of ten participants; “*most therapists”* indicated responses from six to nine; “*half”* denoted five; “*some therapists”* referred to three to four; and “*a few therapists”* applied to one or two participants.

### Trustworthiness

Throughout the analytic process, regular team consultations were held to ensure reflexivity and consistency. The analysis was conducted manually, allowing for close, iterative engagement with the data in keeping with IPA principles. The first and last authors led the initial coding and thematic clustering, while the second author conducted all the interviews. Each team member kept reflexive memos to document personal reactions, emerging insights, and potential biases. Team meetings provided opportunities to compare interpretations, discuss alternative readings of participants' accounts, and refine theme titles and hierarchies. These reflexive discussions enhanced interpretative depth and ensured that the evolving thematic structure accurately represented aspects of participants' experiences. Given that the research team includes practitioners trained in DMT, the analysis was approached with an embodied and relational sensitivity. To manage this positioning, reflexive memoing, ongoing dialogue, and repeated returns to the raw data were employed to ensure that interpretations remained grounded in therapists' own descriptions rather than professional assumptions.^1^

### Findings

The analysis identified two superordinate themes encompassing nine subordinate themes, several of which include finer sub-categories that illustrate nuanced aspects of the therapists' experiences. The first superordinate theme, *The Therapeutic Value of Dyadic Interventions for Parents and Children During Emergency Situations*, comprises five sub-themes describing the multiple relational and regulatory functions of the dyadic frame: (1.1) *Quiet Presence as Support for the Parent*; (1.2) *Working on “Two Parallel Axes” Expands Relational Availability*; (1.3) *The Therapist as an Extension of Parental Qualities*; (1.4) *Initial Motivation and the Parent's Emergent Capacity to Benefit from the Sessions*; and (1.5) *The Therapist's Presence as a Support for Witnessing and Containing the Child's Intensity*. The second superordinate theme, *Why Movement?*, includes four sub-themes that illuminate the distinctive contribution of movement as a medium for nonverbal expression, regulation, and reconnection when words are insufficient: (2.1) *Movement as a Projective Medium*; (2.2) *Embodied Empowerment in the Parent-Child Dyad*; (2.3) *Assessment and Evaluation*; and (2.4) *Meeting Needs: Regression, Rest, and Relational Safety*. Each theme is presented below with supporting quotations that exemplify therapists' lived experiences and interpretations.

**Figure 1:**
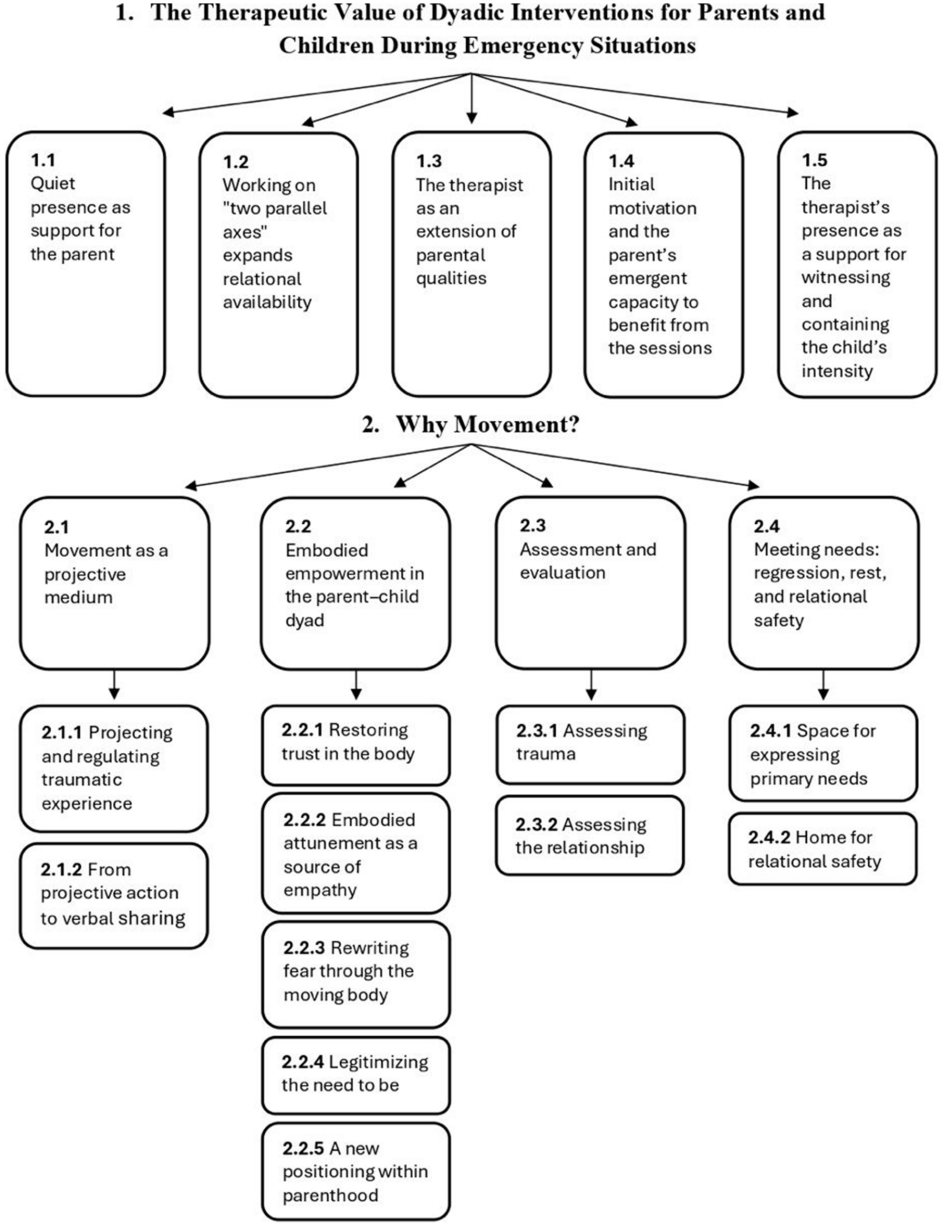
Hierarchical structure of themes identified through Interpretative Phenomenological Analysis (IPA). The diagram illustrates the two superordinate themes and their subordinate themes derived from the therapists' narratives.

Figure 1 presents the hierarchical structure of themes that emerged from the analysis.

**1. The therapeutic value of dyadic interventions for parents and children during emergency situations**

**1.1 Quiet presence as support for the parent**

According to most therapists, the quiet presence of the parent, even without active participation, held significant therapeutic value. The simple act of observing their child engage in free play in the presence of a therapist became a healing experience for parents, who were themselves often in a frozen and anxious state as a result of the traumatic events they had endured. Therapists consistently observed that parents arrived tense and overwhelmed, and that merely sitting alongside their child as the child played gradually softened this tension and supported early regulation. One therapist explained: “*The children were often in a slightly better state than the parents, and the parents needed support in order to be emotionally available to their children.”*

Several therapists described how many parents who initially attended the sessions did so reluctantly, assuming that the intervention was intended only for the child. To illustrate this dynamic, one therapist described the parents' initial stance and how it shifted over time: “*At first, some parents didn't really want to come. They just sat there quietly, not participating, almost as if waiting for it to end. But even that quiet, detached presence had an effect on them. Over time, they softened.”* Another therapist reflected on the internal process that began to unfold for parents within this quietness: “*It's as if just being there, still and quiet, helped them notice their own tension and sense how exhausted they were. Simply being there became nourishing.”*

Across accounts, therapists emphasized that moments of shared quietness often marked the beginning of emotional regulation and reconnection for the parent. Even without words or active engagement, quite presence itself became therapeutic, offering a gentle entry point into the therapeutic process and allowing parents to begin healing alongside their children.

**1.2 Working on “two parallel axes” expands relational availability**

According to most therapists, the dyadic setting offers a dual therapeutic axis: one for the child and one for the parent. When parents use the space to reflect on their emotional experiences, ask questions, or seek guidance, they become more emotionally available, not only to themselves but also to their children. This emotional openness allows the child to step out of a caretaking or reactive role and instead engage from a more authentic, childlike position. One therapist described how a mother expressed relief in having a place to make sense of her own reactions: “*Just having a place where I can ask questions and understand what's happening to me…”* This act of self-reflection, according to all the therapists, enhanced the parent's capacity for co-regulation, which in turn supported the child's emotional regulation: “*The parent's regulation helps the child's regulation.”* Another therapist emphasized the therapist's role in helping both parent and child identify resources for coping, stating: “*Clinician must assist both the parent and the child in accessing resources, calming down, regulating, and releasing tension.”* Through this joint process parents often gain insight into the unconscious influence of their own histories, as one therapist noted: “*During the sessions, parents begin to realize how much their personal history shapes their reactions.”* Therapists repeatedly described how shifts in the parent's and child's availability influenced one another, creating a reciprocal process that unfolded throughout the dyadic encounter. This multidirectional dynamic within the dyadic setting enabled a shift from mere survival to mutual presence, fostering new possibilities for emotional connection and growth.

**1.3 The therapist as an extension of parental qualities**

All therapists referred to the powerful role the therapist plays in reawakening the parent's sense of vitality and emotional engagement with their child. The therapist acts as an extension of the parent's own parental functions, offering the child affirming, soothing, and attuned responses that the parent, in their current state of distress, may struggle to access themselves. By voicing these forms of attuned responsiveness, therapists often enacted modes of parental availability that helped reanimate the parent's own emotional presence. For example, participants described offering supportive verbal responses to parents and children, saying things such as**:** “words of encouragement and comfort”, “expressing admiration for the child's abilities”, and “delight in the child's play and intentions.” These small, embodied moments of verbal attunement served as gentle models of parental responsiveness, illustrating how emotional connection could be re-established even amid acute stress. This therapeutic stance, rich in affective presence, often helped parents reconnect with their own capacity for enjoyment and appreciation of their child. One therapist illustrated this dynamic by saying: “*Through my play with the children, the parents gradually drew closer and opened up, became involved, and a joint encounter was created*.” It is precisely in these moments when the parent witnesses a different, more vital and playful narrative of the child that something new begins to unfold in the relationship. The therapist's embodied engagement becomes a bridge, not only for the child's expression but also for the parent's rediscovery of their own role as a source of joy and connection.

**1.4 Initial motivation and the parent's emergent capacity to benefit from the sessions**

According to most therapists, the majority of the parents initially came to the sessions for the sake of their children. The children often engaged with the space quickly and energetically, displaying an immediate capacity to play, express, and explore. However, as the sessions unfolded, it became increasingly clear that the parents, who had originally positioned themselves as supportive bystanders, were in fact carrying significant emotional needs of their own, often greater than anticipated. Most of the therapists described that as the children settled into play and interaction, the parents were gradually drawn into the therapeutic space, not by deliberate design, but through a kind of emotional pull created by their child's presence and activity. One therapist described: “*At first, the parents sat on the side, almost invisible. But as the sessions went on, you could see them leaning forward, starting to smile, to mirror their child's movement. It was as if something inside them woke up*.” Another therapist reflected: “*Parents often said they came ‘for the child,' but somewhere along the way they realized the space was also for them. Having a few moments to breathe and feel, without needing to protect or function - was profoundly healing*.” What began as a child-focused intervention gradually revealed itself to be a space of healing and connection for the parent as well. It became a place where they could breathe, reflect, and begin to engage with their own internal world. As one therapist noted: “*Through the children, the parents began to engage in something shared.”* This shift allowed the parents to become more emotionally available to themselves and, as a result, to their child.

**1.5 The therapist's presence as a support for witnessing and containing the child's intensity**

According to all the therapists, the presence of the therapist within the parent-child dyad creates the conditions for parents to remain present and emotionally available in moments of stress and helplessness. This shared presence becomes particularly vital when the child exhibits complex behaviors, such as dysregulation, aggression, or emotional withdrawal, that might otherwise overwhelm or confuse the parent. The therapist serves not only as a mediator but as a grounding figure who enables the parent to witness the child's expression without reacting impulsively or interpreting it through a disciplinary lens. One therapist explained: “*Just the fact that the children were playing next to their parents allowed the parents to see how the children were reenacting the trauma through their bodies, without being frightened by it or judging it as inappropriate.”* Another therapist shared: “*The children were engaged in powerful war games. The parents were able to witness their children's intense movements, listen to what was being expressed through them, and become less judgmental.”* This reflective stance, supported by the therapist's presence, opened spaces for meaning-making and reduced the parent's sense of threat. It transformed moments of disconnection into opportunities for understanding, attunement, and renewed connection. These moments of containment often unfolded through movement, small gestures, shared rhythm, or simple bodily presence, that supported both parent and child when words were absent. Building on this, the second superordinate theme, *Why Movement?* examines how embodied expression itself becomes a primary therapeutic medium, fostering communication, regulation, and connection during emergencies.

**2. Why movement?**

All the therapists interviewed consistently emphasized that movement is not merely a therapeutic technique, but a vital medium that allows for expression, connection, stabilization, and transformation, especially when words are inaccessible or inadequate.

**2.1 Movement as a projective medium**

One of the most significant roles of movement in the therapeutic process, as described by all the therapists, is its function as a projective space. Movement enabled children to externalize internal experiences through symbolic, metaphorical, or role-based actions. Therapists described how children “*placed”* feelings, fears or intentions into movement motifs, spatial patterns, gestures, and embodied enactments, allowing inner states to take form outside the body. In this way, one therapist described: “*Movement often opened a path for emotional expression, co-regulation, and meaning-making that felt safer than verbal discourse.“*

**2.1.1 Projecting and regulating traumatic experience**

All of the therapists emphasized that movement allowed children to project and regulate traumatic experience without becoming overwhelmed by premature verbalization. Movement provided a symbolic, nonverbal space in which intense internal states could be externalized into gesture and action, enabling the trauma to be acknowledged without reactivation. Most therapists described how verbal expression, when it appeared, emerged spontaneously. Many referred to this phenomenon as a “*release of speech”*, noting that as one participant explained: “*Words surfaced only after safety, bodily expression, and symbolic projection had been established*. Therapists also highlighted that beginning with the body made it possible to access the child's internal world without imposing narrative demands. As another participant elaborated: “*It's about listening to the most primal form of speech, even when there is no story, which allows us to begin from the child's internal world, rather than from something external or imposed.”* A third therapist emphasized the centrality of nonverbal emergence, noting that for many children: “*Movement enables spontaneous, wordless expression.”* Across accounts, therapists underscored the importance of allowing children to “*tell the story through the body*,” describing this as an essential foundation for regulation and meaning-making in the aftermath of trauma.

A clear example of this projective process occurred when one child, while engaged in a movement-based game involving naming cities, reached a city that frightened him, froze, and announced: “*Stop! We can't move any further.”* His abrupt stillness, experienced as full-bodied halt, externalized the fear associated with that place. The pause functioned as a projective gesture: the child displaced an internal sense of danger into a concrete bodily action, allowing the fear to appear “outside” himself within the protection of play. Therapists understood such halting movements as projective acts in which children cast inner tension or threat into embodied form. Rather than describing fear verbally, the child enacted it through a stopping of the body, marking emotional and physical boundaries inside the safety of the game. These projective movements enabled children to externalize overwhelming sensations into observable actions, offering both containment and the beginning of regulation.

Another therapist described a boy moving in space with large, stretchy fabric, transforming it into a “*trap*” to capture the “*bad guys*” using his body to express powerful, strong movements. The therapist understood this enactment as a projective act in which the child displaced inner tension and a need for control into symbolic, embodied action:

*When children played out these scenes, it was never just “pretend.” You could feel that they were placing something from inside into the movement, the fear, the tension, the need to feel powerful again. They weren't talking about what happened, but their bodies were telling it in a way that was safe for them. The movement became the story, and you could see the child gaining a sense of control as the play unfolded*.

According to several therapists, such symbolic projections were often the child's first step in reorganizing internal experience. By casting sensations, impulses, or narratives into movement, children created enough emotional distance to begin stabilizing and integrating what they felt. In this way, bodily action functioned as a projective bridge: through movement, children could make inner experience visible, shareable, and containable, establishing the groundwork through which verbal meaning could later emerge.

**2.1.2 From projective action to verbal sharing**

While the previous sub-theme focused on how children projected internal experiences into symbolic bodily action, therapists described a sequential process in which such projective movement often created the emotional and embodied conditions necessary for verbal sharing to emerge.

Several therapists emphasized that *only after* children externalized inner states through movement could language begin to surface. To make this progression explicit, therapists frequently described verbal expression as arising “*after the body spoke*,” as one participant articulated it, indicating that speech became possible by the safety and distance created through embodied projection. Rather than beginning with questions or attempts at dialogue, another therapist highlighted that sessions unfolded through action: “*Sessions always began with doing. We started with movement, with play. There was no pressure to talk. Once the child was moving freely, the words came on their own.”* In this account, embodied action supported the child's readiness to share verbally. Another participant added that certain profound expressions surfaced “*only through movement*,” noting: “*Words were born from the intensity of movement. They came after the body had already said something […] Movement carried the first traces of speech, an embodied readiness that preceded verbalization”*.

As children regained physical vitality, therapists often witnessed a parallel reawakening of voice. One therapist observed: “*When her body came back to life, that's when the words came.”* This reanimation was described as a turning point, when the child's internal rhythm became organized enough for symbolic, verbal meaning to emerge. Across these accounts, movement was not a warm-up for speech but the bridge that made speech possible. By projecting sensations, impulses, or fragments of experience into movement, children created the emotional distance needed for expression. For traumatized children, therapists consistently described the same sequence: the pathway to language begins in the body.

**2.2 Embodied empowerment in the parent–child dyad**

**2.2.1 Restoring trust in the body**

All therapists emphasized the importance of helping children and their parents regain a sense of trust, pride, and ownership in their bodies. The therapeutic goal, as described by the therapists, was to restore the sense that the body belongs to the child, responds to their will, and can once again be a source of vitality and self-expression. As one therapist put it: “*My goal was to help him feel that his body belongs to him again, that it listens to him and can also bring him pleasure, not only fear.”* Another therapist described this process as “*helping the child find pride and confidence in what the body can do*.”

A few therapists described facilitating a shift from “*contraction to expansion*”, and from “*passivity to action*”, while others spoke of facilitating a shift from “*stillness to expressive, even ballistic movement*”. One therapist recalled: “*He came in completely folded into himself, sitting in the corner. Slowly he started stretching, jumping, taking up more space. You could see his body filling with life again”*. The invitation was not only to move, but to do so with pleasure and shared playfulness. As another therapist explained: “*We focused on movement that felt good to them, swinging, rolling, running, until their breathing became more natural, regulated, they established a sense of grounding.”* In this sense, therapists described movement as a primary grounding resource in the acute phase, helping children and parents stabilize in their bodies before engaging in deeper emotional work. Through this process, the body is no longer experienced solely as a site of fear or dissociation, but gradually reclaimed as a personal and empowering resource.

**2.2.2 The therapist's embodied attunement as a primary source of empathy**

Most therapists highlighted that their own embodied presence played a central role in supporting children and parents to feel safer and more confident in the therapeutic space. Rather than relying primarily on verbal interpretation, they engaged in a form of embodied listening and response that enacted connection and trust without words. One therapist explained: “*It's about being in dialogue through movement. I adjust my rhythm, my distance, even my breathing, to meet where they are, sometimes just swaying slightly with them until something softens*.” Across accounts, therapists described how these subtle bodily adjustments created a shared emotional field that helped regulate distress and invited both parents and children into a sense of relational safety. Another reflected: “*The body knows how to listen when the mind is overwhelmed. When I move with the child or mirror a parent's small gesture, it tells them they're not alone.”*

Through such movement-based responsiveness, therapists became a live example nonverbal dialogue. They described attending to cues such as physical proximity, intensity, rhythm, and tempo as signals of each person's emotional state and relational needs. By adjusting their body in relation to the dyad, they offered a model of sensitivity, respect for boundaries, and stable presence. In doing so, they helped parent and child experience a felt sense of being seen and understood, which in turn supported their confidence and capacity to engage more fully in the therapeutic process.

**2.2.3 Rewriting fear through the moving body**

Many therapists spoke about movement as a medium through which children can safely explore experiences of control and power in the aftermath of trauma. Within a protected therapeutic space, movement allowed children to reclaim agency, not through explanation, but through playful and embodied action. One therapist recalled a 2-year-old who repeatedly exited the room only to return with a loud roar, “*like a lion*.” Each re-entry, filled with intensity and theatrical flair, asserted control in a space that welcomed this presence. “*There was joy in it*,” the therapist reflected, “*a connection to vitality, to childhood, to playfulness through the body.”* The therapist responded with enthusiasm rather than correction: “*Wow, what a surprise!”* She viewed this not as a behavioral problem, but as a healthy, embodied response to an uncontrollable reality. By welcoming the child's impulse to enact and reverse roles of fear and power, the therapist supported a bodily reworking of frightening experiences that might otherwise be pathologized.

**2.2.4 Legitimizing the need to be seen**

All of the therapists spoke about moments in which children expressed a deep need for visibility in the therapeutic space. This need was often communicated indirectly through bodily gestures, positioning, or symbolic acts. One therapist observed how, several times during the session, a 4-year old child stood up on a chair, as if declaring: “*I'm here!”* The mother, however, frequently lost focus or became distracted. Sensing the child's silent plea for attention, the therapist chose to mirror his gesture with warmth and humor: “*Wow, you're so tall up there! I see you.”* She later reflected: “*There was playfulness and humor in it. It was an expression of need without words.”* For this therapist, the child's elevated posture functioned as a nonverbal statement of his wish to be noticed, and her response offered a moment of recognition without confrontation. In this way, movement and positioning became a language through which the child's need to be seen could be acknowledged. The therapist's playful mirroring granted legitimacy to that need and created a small but significant experience of visibility and connection.

**2.2.5 A new positioning within parenthood**

Within the broader process of reclaiming bodily and relational empowerment, therapists described how movement enabled parents to experience themselves differently in relation to their children. Most therapists emphasized that movement-based work offered parents an opportunity to shift from crisis-driven, managerial stance into a more present, playful, and emotionally available mode of relating. As one therapist explained: “*Movement work allows us to stay with an issue that is expressed through the body, rather than having to treat, manage, or educate.”* This shift opened the door to a different kind of relational experience, one in which the parent is not locked into the role of the responsible, worried adult. Another therapist reflected on the emotional tone that movement invites: “*Movement brings joy and playfulness to the parent, in contrast to the usual positioning within the responsible, concerned parental role.”* Through such embodied engagement, parents reconnected with aspects of themselves that had been muted by fear, exhaustion, or the demands of coping with crisis. In this sense, movement became not only a medium of expression for the child, but also a gateway to relational renewal for the parent, offering moments of lightness, spontaneity, and shared presence that therapists described as central to both empowerment and a gradual return to more “normal,” everyday parent–child interaction.

**2.3 Assessment and evaluation**

**2.3.1 Movement as a tool for assessing trauma**

All therapists referred to the body, and specifically to movement patterns, as a key indicator of trauma. Trauma, they observed, often became visible through restricted, disorganized or overly intense movement qualities. Shifts in movement—in variability, fluidity, rhythm and freedom of flow—served as important markers of therapeutic change. As one therapist noted: “*Some children responded to the trauma they experienced with very intense movement, which slowly settled into more regulated, socio-dramatic movement play.”* Therapists emphasized that these observable changes were shared with the parents to support understanding and reflection: “*Over time, the body became more relaxed. It's important to show that to the parents. The body is a language.”* Movement also offered a structured way to assess regulatory and relational cues: “*It's important to examine avoidance in eye contact, difficulties in synchronization, in shared attention, in intensity, in spatial distance.”* These subtle embodied markers provided therapists with crucial insights into the child's inner world, their regulatory capacity, and shifts in emotional functioning across the course of the intervention.

**2.3.2 Movement as a tool for assessing the relationship**

According to all therapists, movement also revealed the current state of the parent–child relationship. When words were limited or unavailable, nonverbal interaction patterns, i.e. proximity, rhythm, synchrony, posture, and shared attention, offered rich diagnostic insight into the emotional climate of the dyad. As one therapist explained:

*There's implicit dyadic knowledge. Watching the nonverbal relationship: does the child sit with their back to the parent, close, far away? […] When a topic emerges, I offer a prop and invite them into play. Through that, they encounter the complexity of the experience, and then we can speak from a different place*.

Here, the therapist uses the prop as an assessment tool: the way parent and child enter, avoid, or negotiate movement play reveals patterns of connection, tension, and relational readiness. Another therapist described a moment of assessing relational comfort: “*A girl sits on her father's lap, but he feels uncomfortable. I offer them a stretchy fabric and suggest trying different distances like sitting closer, farther apart, so they can feel how each position makes them feel”*. These guided, embodied adjustments allowed both therapist and parent to observe patterns of proximity, discomfort, and mutual regulation, opening a pathway to deeper understanding of the relationship with the child. In this sense, the shifts in spatial distance functioned not only as interventions but also as diagnostic probes, enabling the therapist to assess the dyad's regulatory patterns, boundaries, and comfort levels while allowing parents to witness these relational dynamics directly.

**2.4 Meeting needs: regression, rest, and relational safety**

**2.4.1 Space for expressing primary needs**

Most therapists reported that movement provided a channel for children to express early developmental needs such as holding, containment, cradling, rest, and self-soothing. Through movement, children could access preverbal experiences of attachment and relational safety that were difficult to reach verbally. As one therapist explained: “*Movement offers legitimacy and pleasure in being small and needing care.” One* example involved a 4-year-old girl who had reverted to bedwetting, was restless, tearful, and spoke very little. The mother was anxious, and the father emotionally distant. The therapist described: “*The child wanted to crawl and be a baby again. I invited the mother to see her baby, that's what the body was expressing.”* In another case, the therapist described a moment when both an infant and an older sibling communicated deep needs for soothing:

*I picked up the baby and sang to her. The older child watched in wonder and then asked to lie down like a baby. I said to him, ‘It's okay—you're allowed to rest.' A week later, the mother reported that he was noticeably more relaxed*.

Across accounts, therapists emphasized that these moments of regression were not treated as pathology, but as natural responses to trauma and disconnection. The movement-based space allowed such needs to surface safely, making room for embodied experiences that words alone could not hold.

**2.4.2 A home for relational safety**

In the aftermath of trauma, both child and parent often carry heightened emotional tension. Parents may feel anxious, depleted or emotionally shut down, while children struggle with regulation and uncertainty. According to all therapists, movement-based interaction created a “relational home”, a safe, embodied space in which these emotional dynamics could be held without judgment or overwhelm. Within this space, regression, rest, and emotional closeness could emerge naturally, supporting both members of the dyad. The body becomes a shared language through which parent and child can meet again, not as dysregulated caregiver and struggling child, but as two people engaging in a mutual process. One therapist described this process:

*At first, the parents were stiff, watching from the side. But when the child began moving, they slowly joined. Sometimes they sat together on the floor, breathing at the same pace. You could feel the room settle. It became a place where they could meet again, not as anxious parent and frightened child, but simply as two bodies finding each other*.

Another therapist explained how shared movement restored relational rhythm: “*When the parent moved with the child, even a small sway, something shifted. The child relaxed, and the parent softened. The movement gave them a safe place to reconnect.”* A third therapist echoed this pattern, noting the relational opening created through movement: “*Over time, parents became more willing to join their children in movement and to experience the intensity together with them.”* Across accounts, these shared embodied experiences marked turning points in which the relationship itself could be felt, regulated, and redefined through the body rather than through words alone. In this way, movement offered a preverbal foundation for relational safety, becoming the ground upon which connection could be rebuilt after trauma.

## Discussion

This study explored psychotherapists' interpretations of dyadic, movement-based interventions in emergency settings with parents and children during times of crisis and displacement. Two major thematic domains emerged: (1) the therapeutic value of dyadic intervention for parents and children in emergency settings, and (2) the centrality of movement as a transformative and diagnostic medium in trauma work.

In the following discussion, the five sub-themes identified under the overarching domain “*The Therapeutic Value of Dyadic Interventions During Crisis and Emergency Situations”* are integrated into three meta-themes that capture higher-order conceptual insights: (1) the therapist's active role in crisis and embodied vitality as a bridge to parental reconnection, (2) dyadic work as a two-axis process, and (3) beginning with the child as a way to gradually involve the parent in therapy. These meta-themes represent a synthesis of the findings at a broader interpretive level, aiming to articulate the underlying therapeutic principles of dyadic, movement-based work in emergencies.

### The therapeutic value of dyadic interventions for parents and children during crisis and emergency situations

The findings highlight the unique potential of dyadic interventions to support both emotional regulation and relational presence of parent and child in emergency contexts. Rather than positioning the child as the sole “identified patient,” (Pantone, 2000), the dyadic approach establishes a shared therapeutic space in which both members of the relationship are seen, supported, and offered the opportunity for transformation. Unlike conventional dyadic therapy, which often emerges in response to relational difficulties or developmental challenges, the interventions discussed here are situated within external crises of war and displacement. In such contexts, distress is not primarily rooted in the parent–child relationship itself, but arises from overwhelming environmental chaos and contextual threats (Sim et al., 2018). Accordingly, the therapeutic focus shifts from resolving relational pathology and repairing internal relational dynamics to restoring emotional vitality and presence amid conditions of survival.

Therapists emphasized the power of the parent's physical and emotional presence, even when silent. This aligns with attachment-based frameworks (Bowlby et al., 1992) and models of co-regulation (Feldman, 2007), which underscore the regulatory potential of proximity and attuned attention. In moments of fear or immobilization, the joint presence of parent and therapist offered the child a sense of anchoring. The parent's witnessing presence provided a sense of safety enabled the child's emotional expression, while allowing the parent to reconnect from a renewed perspective.

In trauma contexts, presence is not passive. It enables a form of nonverbal mirroring that affirms the child's inner experience and supports the parent in shifting from anxious vigilance to engaged recognition (Hadar et al., 2025). This dynamic is consistent with models of right-brain attunement and mutual regulation that emphasize the role of embodied presence in restoring emotional organization following stress (Beebe and Lachmann, 2002; Stern, 2010). Parents moved from fear to curiosity, reconnecting with their child not as a problem but as a source of vitality and relational meaning. Parents were gradually able to reconnect emotionally, both with themselves as caregivers and with their child as an agent of vitality and relational meaning. These observations suggest that dyadic work in crisis stabilizes the child, while facilitating parental re-engagement (Lieberman and Van Horn, 2011; Masten and Narayan, 2012), highlighting the distinctive therapeutic stance required in times of crisis (Hadar et al., 2025).

#### The therapist's active role in crisis: embodied vitality as a bridge to parental reconnection

A central distinction in emergency dyadic therapy is the therapist's heightened embodied activity during acute crisis. Therapists temporarily stepped into parental functions that parents, due to trauma or depletion, could not access. Unlike conventional dyadic approaches, where therapist over-involvement might be seen as disempowering or intrusive (Fraiberg et al., 1975; Lieberman and Van Horn, 2011), this stance was understood as a short-term, adaptive response that supported, rather than replaced, the parent's own capacity for attunement and engagement.

Therapists used gestures of play, rhythm, and physical presence to offer affective responsiveness to the child while modeling engagement for the parent. This embodies the regulatory power of nonverbal co-regulation and right-brain communication (Ogden et al., 2006; Schore, 2003). Once parents witnessed their child being met with warmth and attuned presence, they were drawn into participation, suggesting that embodied activity in emergencies may facilitate re-engagement more rapidly than traditional models.

#### Dyadic work as a two-axis process

Therapists described the therapeutic encounter as unfolding along two interdependent axes, one oriented toward the child, the other toward the parent, reflecting relational trauma models where each member's well-being is interconnected (Lieberman et al., 2005). When parents were supported in processing their own distress, they became more emotionally available, enabling more effective co-regulation. This shift in parental presence allowed children to re-inhabit developmentally appropriate modes of play and expression rather than compensating through premature caregiving or withdrawal. These observations echo findings that a more regulated parent supports a more regulated child (Tronick, 2007), especially salient when the parent's nervous system is overwhelmed.

This two-axis process mirrors evidence that children's adjustment during displacement improves primarily through strengthening caregiver regulation and emotional capacity (Sim et al., 2018). Rather than fragmenting support, the dyadic frame fosters a shared field of emotional regulation and co-recovery, where both participants benefit through mutual resonance and influence.

#### Starting with the child: a gradual therapeutic engagement of the parent

Although many parents initially entered the sessions assuming they were primarily intended for their child, therapists observed that, over time, the emotional and relational dynamics shifted. The child's expressive movement gradually drew parents into a shared emotional field, revealing their own needs for regulation and connection. Parents were engaged through the natural relational pull of the child's movement, echoing models where embodied cues and shared rhythms create connection (Kestenberg and Buelte, 1977; Stern, 2010). In this context, the child often served as a regulatory anchor, inviting the parent into moments of shared presence and reintroducing elements of playfulness, spontaneity, and emotional engagement. This bidirectional process facilitates the child's regulation while enabling parents to access internal resources and relate from a renewed, more flexible stance, reweaving connection in trauma's aftermath.

### Why movement? The role of movement in dyadic therapeutic work during crisis

The second major theme highlights movement as a foundational therapeutic language, particularly vital in crisis contexts where verbal expression is often limited or inaccessible. In these settings, movement served multiple therapeutic functions, such as a projective medium, a channel for regulation and expression, a diagnostic tool, and a relational bridge between parent and child. Movement weaves together these functions, enabling therapeutic transformation across the dyadic process.

#### Movement as a projective, regulating, and empowering medium

Movement enabled children to safely externalize complex emotions and facilitated regulation. Patterns such as contraction followed by expansive release signaled a shift from immobilization to vitality (Amighi et al., 1999), keeping the child within a window of tolerance. These moments of renewed liveliness resonate with Stern's (2010) concept of vitality forms, which describe the dynamic qualities of movement that underpin relational experience. Such shifts often included ballistic movement (Shahar-Levy, 2001) – spontaneous, forceful gestures signifying release of stored tension. These embodied experiences restored a sense of control and agency in children who had felt helpless or frozen. In reclaiming bodily agency, children no longer experienced their bodies as passive or threatening. Instead, through exploratory movement, they rediscovered play, boundary-testing, and self-expression. This aligns with research showing DMT supports emotional regulation and trauma processing across diverse populations (Betty, 2013; Devereaux and Harrison, 2022; Grasser et al., 2019; Harris, 2007).

This reclaiming of bodily agency was not limited to children. Parents, too, were often drawn into moments of movement, rediscovering joy and co-regulation alongside their children. Movement allowed parents to step out of rigid caretaker roles and meet their children from mutual enjoyment. This shift from “managing” to “meeting” the child created new pathways for restoring connection, co-regulation, and mutual transformation within the parent-child dyad.

This parallels Tronick's (2007) Still-Face paradigm, where ruptures in attunement are repaired through renewed emotional presence. Therapists helped parents move from frozen inaction to re-engaged responsiveness through embodied co-movement, where the child's spontaneous vitality catalyzed the parent's release. Taken together, the findings indicate that the dyadic setting constitutes a bidirectional field of healing, in which therapeutic gains occur not only in the child's regulation but in the parent's vitality and emotional re-engagement.

Although therapists worked with families from diverse cultural and religious communities, their narratives contained almost no explicit reference to cultural meanings surrounding movement, touch, or emotional expression. One interpretation of this relative silence is that it reflects the immediacy and intensity of the emergency context, in which therapists focused primarily on basic regulation, safety, and stabilizing presence. In such acute conditions, shared human needs for protection and connection may temporarily overshadow culturally specific interactional patterns. This interpretation highlights the importance of examining how cultural frameworks re-emerge or shift once the immediate crisis phase stabilizes, and how embodied dyadic processes are negotiated within and across different cultural groups.

#### Movement as witnessing and diagnostic tool

Therapists described movement as a site of mutual witnessing, where they and parents could attune to the child's inner world through nonverbal cues. This mirrors *Authentic Movement* principles (Payne, 2024), where witnessing spontaneous movement cultivates empathy and relational presence. This witnessing became transformational: it created moments of embodied recognition, an “I see you”, that transcended verbal articulation. For parents, this allowed a softening of defenses and a shift toward experiencing their child's inner world with greater compassion and emotional access.

Movement also served as a diagnostic lens. Therapists observed micro-patterns in posture, proximity, synchrony, and rhythm to assess regulatory capacity and attachment dynamics (Beebe and Lachmann, 2002). Patterns of tension, withdrawal, or misattunement guided real-time adaptations and informed therapeutic strategy. This use of embodied observation is consistent with dyadic interaction research showing that subtle nonverbal cues, such as shifts in rhythm, affective timing, or spatial orientation, reflect underlying regulatory processes and relational expectations (Feldman, 2007; Stern, 2010).

Movement enabled relational change. Structured and spontaneous movement allowed dyads to explore new forms of closeness, separation, and responsiveness. Such experiential renegotiation aligns with models of embodied psychotherapy, which propose that relational change occurs not only through insight but through new sensorimotor experiences that revise implicit relational memories (Amighi et al., 1999; Frank and La Barre, 2011). These shared embodied experiences enabled new relational narratives grounded in play and mutual exploration rather than fear or control.

#### Embodied attunement: the therapist's presence as intervention

Underpinning this work was the therapist's own embodied presence as a central mode of therapeutic communication. Rather than relying primarily on verbal interventions or behavioral strategies, therapists engaged in bodily listening, attuning to shifts in proximity, intensity, and movement quality as core emotional signals. This reflects right-brain communication models (Schore, 2003) positioning the therapist's body as a medium for co-regulation, attunement and containment (Vulcan, 2009, 2016). Through physical presence and relational movement, therapists modeled embodied safety and emotional availability. In the aftermath of trauma, when verbal and symbolic communication can be fragile or inaccessible, this embodied relationality served as a crucial bridge. It enabled children and parents to feel recognized and met on a bodily level, often before verbal expression became possible.

#### Accessing preverbal needs and creating a home for the relationship

Movement opened access to early developmental needs that resurface in the wake of trauma, a need for rest, containment, soothing, and regression. Therapists described how children and parents alike signaled needs for holding or soothing through posture, gesture, and proximity. Rather than pathologizing these expressions, therapists recognized them as natural responses to disconnection and emotional exhaustion. These moments of embodied regression were often marked as turning points in the therapeutic process.

The movement space became a relational “home,” where parent and child could meet beyond the labels of “dysregulated child” and “distressed caregiver.” Within this space, relational repair occurred not through interpretation or insight, but through co-regulated presence, shared rhythm, and embodied availability. Movement made room for a quality of meeting that was physical, emotional, and deeply human. In this respect, the findings align with research on dyadic arts-based therapies in displacement contexts, which similarly highlight how preverbal, sensory, and rhythmic experiences can support early attachment needs when verbal communication is compromised (Hadar et al., 2025). These parallels suggest that across creative arts modalities, embodied and regression-allowing experiences may offer a foundational route to relational repair when both parent and child are operating with reduced emotional capacity.

### Clinical implications: beyond movement-based interventions

The findings of this study offer clinical insights that extend beyond the field of movement-based interventions. They underscore the therapeutic value of embodied presence and position the body as both a source of clinical knowledge and a central instrument of healing. For all therapists working with families in crisis, this research highlights attending to preverbal and bodily dimensions of distress.

Key implications include recognizing both child and parent as clients requiring emotional holding, validating shared presence as a regulatory force, and using movement, play, and embodied attunement to facilitate connection. In emergency contexts where trauma is ongoing and verbal capacities are often compromised, movement and relational presence may become primary therapeutic interventions, a direct route to reconnection and repair.

The therapist's capacity to move, play, and act without waiting for words may reopen the parent–child relationship. In these moments, the therapist does not simply observe or facilitate but participates in the creation of a shared rhythm of recovery, one that begins in the body, and only later may find its way into other forms of language. Recent client-centered research (Klapisch et al., 2025) reinforces these findings, showing how nonverbal resonance, attunement, and embodied enactment support trauma recovery from the client's perspective. This convergence between therapist and client perspectives underscores a core clinical principle: actively shaping the therapeutic space as a co-regulated, embodied environment rather than relying solely on verbal or insight-oriented methods. For clinicians, this means attending to the embodied interactions of both members of the dyad, intervening through shared movement and rhythm, and supporting the co-construction of a regulated relational field. In this way, mutual embodied engagement becomes a core clinical pathway for restoring vitality and connection within the family system.

## Limitations and directions for future research

The findings of this study are derived exclusively from therapists' narratives and interpretations. As such, they represent therapists' professional meaning-making rather than direct accounts from parents or children. This single-perspective design offers valuable insight into the therapists' lived experience, yet it does not incorporate the lived perspectives of the families themselves. Future studies would benefit from including multi-perspective data, gathering narratives from therapists, parents, and children, to deepen understanding of the dyadic processes identified here.

In addition, the findings suggest that the dyadic setting constitutes a bidirectional field of healing, with therapeutic gains emerging not only in the child's regulation but also in the parent's vitality and emotional re-engagement. Future research is needed to examine the mechanisms through which parental transformation contributes to sustained post-traumatic growth for the family as a whole.

Cultural factors present another area for further exploration. As noted in the discussion, therapists did not reference cultural meanings surrounding movement, touch, or emotional expression. Although this may reflect the immediacy of crisis conditions, cultural frameworks likely shape embodied interaction in important ways. Future research should investigate how dyadic, movement-based interventions unfold across diverse cultural, ethnic, and refugee contexts.

Finally, this study used descriptive rather than formal movement-analysis terminology to prioritize accessibility and to remain close to the therapists' lived meaning-making. Future research could complement this interpretive approach with systematic coding of movement qualities to deepen understanding of embodied change processes across cultural and clinical contexts. Studies could incorporate video-based movement analysis to capture micro-relational processes such as synchrony, rupture, misattunement, and repair.

## Conclusion

This study offers an in-depth perspective on how psychotherapists, working under acute conditions in emergency contexts, engage in embodied, dyadic interventions to support regulation, reconnection, and relational repair. Although based solely on therapists' narratives, the findings illuminate how therapists mobilize presence, vitality, and relational witnessing, often stepping beyond traditional models of reflective neutrality, to meet the urgent needs of parents and children in crisis. Movement emerged not only as a medium for trauma expression, but also as a diagnostic lens, a relational bridge, and a developmental catalyst, that enabled therapeutic processes rarely accessible through verbal means. These insights underscore the value of body-informed, relationally attuned approaches in emergency care and highlight the dyadic setting as a bidirectional field of healing, where therapeutic change unfolds through reciprocal processes between parent and child. Taken together, the findings illustrate how embodied dyadic work can restore connection and emotional vitality at moments when families face profound disruption.

## Data Availability

The datasets presented in this article are not readily available due to the sensitive nature of qualitative interview data, only anonymized excerpts are available within the manuscript. Further details can be requested from the corresponding author. Requests to access the datasets should be directed to Maya Vulcan, mvulcan@staff.haifa.ac.il.

## Footnotes

1 Methodological note: To ensure accessibility for a multidisciplinary readership, movement was described throughout the study using communicative and clinically descriptive language rather than specialized analytic frameworks such as the Kestenberg Movement Profile (Amighi et al., 1999) and Emotorics (Shahar-Levy, 2016). This decision reflected the study's phenomenological orientation and its focus on therapists' subjective meaning-making rather than movement categorization. The analytic emphasis was therefore placed on the *therapeutic function and lived experience* of movement, as conveyed by the therapists, rather than on formal movement analysis.

## References

[B1] Amighi J. K. Lewis P. Loman S. Sossin K. M. (1999). Meaning of Movement. Amsterdam: Gordon and Breach.

[B2] Arega N. T. (2023). Mental health and psychosocial support interventions for children affected by armed conflict in low- and middle-income countries: a systematic review. Child Youth Care Forum 52, 1431–1456. doi: 10.1007/s10566-023-09741-0 PMC9990564 37360764

[B3] Azoulay B. Orkibi H. (2018). Helpful and hindering factors in psychodrama field training: a longitudinal mixed methods study of student development. Front. Psychol. 9, 1–10. doi: 10.3389/fpsyg.2018.00196 29515504 PMC5826287

[B4] Bauch N. G. (2022). Art therapy with refugee and asylum-seeking children and their parents: preliminary findings of a thorough literature review. Psychol. Appl. Trends 245–249. doi: 10.36315/2022inpact058

[B5] Baumgarten L. G. Johansen M. Winther H. (2022). Holistic movement activities with refugee families: the importance of attachment processes. Body Mov. Dance Psychother. 18, 4–21. doi: 10.1080/17432979.2022.2148743

[B6] Beebe B. Lachmann F. (2002). Organizing principles of interaction from infant research and the lifespan prediction of attachment: application to adult treatment. J. Infant Child Adolesc. Psychother. 2, 61–89. doi: 10.1080/15289168.2002.10486420

[B7] Betty A. (2013). Taming tidal waves: a dance/movement therapy approach to supporting emotion regulation in maltreated children. Am. J. Dance Ther. 35, 39–59. doi: 10.1007/s10465-013-9152-3

[B8] Bowlby J. Ainsworth M. Bretherton I. (1992). The origins of attachment theory. Dev. Psychol. 28, 759–775. doi: 10.1037/0012-1649.28.5.759

[B9] Bürgin D. Anagnostopoulos D. Vitiello B. Sukale T. Schmid M. Fegert J. M. (2022). Impact of war and forced displacement on children's mental health—multilevel, needs-oriented, and trauma-informed approaches. Eur. Child Adolesc. Psychiatry 31, 845–853. doi: 10.1007/s00787-022-01974-z 35286450 PMC9209349

[B10] Burkhart K. Agarwal N. Kim S. Neudecker M. Ievers-Landis C. E. (2023). A scoping review of trauma-informed pediatric interventions in response to natural and biologic disasters. Children 10, 2–26. doi: 10.3390/children10061017 37371249 PMC10297269

[B11] Cicchetti D. Toth S. L. (2009). The past achievements and future promises of developmental psychopathology: the coming of age of a discipline. J. Child Psychol. Psychiatry 50, 16–25. doi: 10.1111/j.1469-7610.2008.01979.x 19175810 PMC3531893

[B12] Cohen E. Chazan S. Lerner M. Maimon E. (2010). Posttraumatic play in young children exposed to terrorism: an empirical study. Infant Ment. Health J. 31, 159–181. doi: 10.1002/imhj.20250 28543328

[B13] Cohen E. Gadassi R. (2018). The function of play for coping and therapy with children exposed to disasters and political violence. Curr. Psychiatry Rep. 20, 1–7. doi: 10.1007/s11920-018-0895-x 29623498

[B14] Crooks A. Mensinga J. (2021). Body, relationship, space: dance movement therapy as an intervention in embodied social work with parents and their children. Aust. Soc. Work 74, 250–258. doi: 10.1080/0312407X.2020.1861315

[B15] Devereaux C. Harrison L. (2022). “Body, brain, and relationship: dance/movement therapy and children with complex trauma,” in Dance/Movement Therapy for Trauma Survivors, eds. R. Dieterich-Hartwell and A. M. Melsom (Abingdon: Routledge), 83–100.

[B16] Dieterich-Hartwell R. Goodill S. Koch S. (2020). Dance/movement therapy with resettled refugees: a guideline and framework based on empirical data. Arts Psychother. 69:101664. doi: 10.1016/j.aip.2020.101664

[B17] Dieterich-Hartwell R. Haen C. Kaimal G. Koch S. Villanueva A. Goodill S. (2021). Developing movement experiences with refugees to the United States who have undergone trauma. Int. J. Migr. Health Soc. Care 17, 75–91. doi: 10.1108/IJMHSC-04-2020-0036

[B18] Feldman R. (2007). Parent–infant synchrony and the construction of shared timing; physiological precursors, developmental outcomes, and risk conditions. J. Child Psychol. Psychiatry 48, 329–354. doi: 10.1111/j.1469-7610.2006.01701.x 17355401

[B19] Fonagy P. Gergely G. Jurist E. L. Target M. (2002). Affect Regulation, Mentalization and the Development of the Self . New York, NY: Karnac Books.

[B20] Fraiberg S. Adelson E. Shapiro V. (1975). Ghosts in the nursery: a psychoanalytic approach to the problem of impaired infant-mother relationships. J. Am. Acad. Child Psychiatry 14, 1387–1422. doi: 10.1016/S0002-7138(09)61442-4 1141566

[B21] Frank R. La Barre F. (2011). The First Year and the Rest of Your Life: Movement, Development, and Psychotherapeutic Change. Abingdon: Routledge.

[B22] Gavron T. Snir S. Berkovsky Y. Azoulay S. Dor L. Franko N. . (2024). Community art therapy (CAT): learning from art therapy graduate students' perceptions. Int. J. Art Ther. 30, 1–10. doi: 10.1080/17454832.2024.2317214

[B23] Gilbert R. Abel M. R. Vernberg E. M. Jacobs A. K. (2021). The use of psychological first aid in children exposed to mass trauma. Curr. Psychiatry Rep. 23, 1–9. doi: 10.1007/s11920-021-01270-8 34232405

[B24] Grasser L. R. Al-Saghir H. Wanna C. Spinei J. Javanbakht A. (2019). Moving through the trauma: dance/movement therapy as a somatic-based intervention for addressing trauma and stress among Syrian refugee children. J. Am. Acad. Child Adolesc. Psychiatry 58, 1124–1126. doi: 10.1016/j.jaac.2019.07.007 31348987

[B25] Hadar T. Slabu L. Franco F. Fedotiuk T. Coombes E. (2025). Music with displaced dyads: Ukrainian parents' perspectives on a music therapy group. J. Loss Trauma 1–26. doi: 10.1080/15325024.2025.2556425

[B26] Harris D. A. (2007). Dance/movement therapy approaches to fostering resilience and recovery among African adolescent torture survivors. Torture 17, 134–155. 17728491

[B27] Kamali M. Munyuzangabo M. Siddiqui F. J. Gaffey M. F. Meteke S. Als D. . (2020). Delivering mental health and psychosocial support interventions to women and children in conflict settings: a systematic review. BMJ Glob. Health 5, 1–16. doi: 10.1136/bmjgh-2019-002014 32201624 PMC7073823

[B28] Kedem D. Regev D. (2021). Parent-child dance and movement therapy (PCDMT): mothers' subjective experiences. Body Mov. Dance Psychother. 16, 136–149. doi: 10.1080/17432979.2021.1883740

[B29] Kestenberg J. S. Buelte A. (1977). Prevention, infant therapy, and the treatment of adults. 1. Toward understanding mutuality. Int. J. Psychoanal. Psychother. 6, 339–367. 914445

[B30] Klapisch O. Gueta K. Shlomi I. (2025). Therapeutic body resonance: pathways to recovery through enactment for childhood sexual abuse survivors. Body Mov. Dance Psychother. 20, 268–286. doi: 10.1080/17432979.2025.2496682

[B31] Kvale S. (2007). Doing Interviews. Thousand Oaks, CA: Sage Publications.

[B32] Lieberman A. F. Padrón E. Van Horn P. Harris W. W. (2005). Angels in the nursery: the intergenerational transmission of benevolent parental influences. Infant Ment. Health J. 26, 504–520. doi: 10.1002/imhj.20071 28682485

[B33] Lieberman A. F. Van Horn P. (2011). Psychotherapy with Infants and Young Children: Repairing the Effects of Stress and Trauma on Early Attachment. New York, NY: Guilford Press.

[B34] Llewelyn S. P. Elliott R. Shapiro D. A. Hardy G. Firth-Cozens J. (1988). Client perceptions of significant events in prescriptive and exploratory periods of individual therapy. Br. J. Clin. Psychol. 27, 105–114. doi: 10.1111/j.2044-8260.1988.tb00758.x 3395733

[B35] Malchiodi C. A. (2020). Trauma and Expressive Arts Therapy: Brain, Body, and Imagination in the Healing Process. New York, NY: Guilford Publications.

[B36] Masten A. S. Narayan A. J. (2012). Child development in the context of disaster, war, and terrorism: pathways of risk and resilience. Annu. Rev. Psychol. 63, 227–257. doi: 10.1146/annurev-psych-120710-100356 21943168 PMC5858878

[B37] Montague J. Phillips E. Holland F. Archer S. (2020). Expanding hermeneutic horizons: working as multiple researchers and with multiple participants. Res. Methods Med. Health Sci. 1, 25–30. doi: 10.1177/2632084320947571

[B38] Nocon A. Eberle-Sejari R. Unterhitzenberger J. Rosner R. (2017). The effectiveness of psychosocial interventions in war-traumatized refugee and internally displaced minors: systematic review and meta-analysis. Eur. J. Psychotraumatol. 8:1388709. doi: 10.1080/20008198.2017.1388709 29163868 PMC5687794

[B39] Ogden P. Pain C. Fisher J. (2006). A sensorimotor approach to the treatment of trauma and dissociation. Psychiatr. Clin. North Am. 29, 263–279. doi: 10.1016/j.psc.2005.10.012 16530597

[B40] Pantone P. J. (2000). Treating the parental relationship as the identified patient in child psychotherapy. J. Infant Child Adolesc. Psychother. 1, 19–37. doi: 10.1080/15289168.2000.10486331

[B41] Payne H. (2024). Relational integrative psychotherapy and the discipline of authentic movement. Am. J. Dance Ther. 46, 34–51. doi: 10.1007/s10465-023-09394-5

[B42] Perryman K. Blisard P. Moss R. (2019). Using creative arts in trauma therapy: the neuroscience of healing. J. Ment. Health Couns. 41, 80–94. doi: 10.17744/mehc.41.1.07

[B43] Rependa S. L. Goldstein L. Fergus K. Watson E. Lawford J. Muller R. T. (2024). Caregiver engagement and treatment response in child trauma therapy: a qualitative analysis. J. Fam. Soc. Work 27, 43–59. doi: 10.1080/10522158.2024.2420922

[B44] Schaeffer A. J. Cornelius-White J. H. D. (2021). Qualitative studies on body-based interventions for refugees: a meta-synthesis. Body Mov. Dance Psychother. 16, 267–285. doi: 10.1080/17432979.2021.1893810

[B45] Schore A. N. (2003). Affect Regulation and the Repair of the Self . New York, NY: Norton.

[B46] Schottelkorb A. A. Doumas D. M. Garcia R. (2012). Treatment for childhood refugee trauma: a randomized, controlled trial. Int. J. Play Ther. 21, 57–73. doi: 10.1037/a0027430

[B47] Shahar-Levy Y. (2001). The function of the human motor system in processes of storing and retrieving preverbal, primal experience. Psychoanal. Inq. 21, 378–393. doi: 10.1080/07351692109348942

[B48] Shahar-Levy Y. (2016). “Emotorics: a psychomotor model for the analysis and interpretation of emotive motor behavior,” in The Art and Science of Dance/Movement Therapy: Life is Dance (2nd ed.), eds. S. Chaiklin and H. Wengrower (Abingdon: Routledge), 261–287.

[B49] Shakarov I. Regev D. Snir S. Orkibi H. Adoni-Kroyanker M. (2019). Helpful and hindering events in art therapy as perceived by art therapists in the educational system. Arts Psychother. 63, 31–39. doi: 10.1016/j.aip.2019.03.005

[B50] Shay D. Navon Y. Blank C. Shavit Y. (2024). Young Children And Their Parents During The War (Taub Center Early Childhood Research Series, Research Paper No. 19). Jerusalem: Taub Center for Social Policy Studies in Israel.

[B51] Sim A. Fazel M. Bowes L. Gardner F. (2018). Pathways linking war and displacement to parenting and child adjustment: a qualitative study with Syrian refugees in Lebanon. Soc. Sci. Med. 200, 19–26. doi: 10.1016/j.socscimed.2018.01.009 29355827

[B52] Slade A. (2005). Parental reflective functioning: an introduction. Attach. Hum. Dev. 7, 269–281. doi: 10.1080/14616730500245906 16210239

[B53] Smith J. A. Flowers P. Larkin M. (2009). Interpretative Phenomenological Analysis: Theory, Method and Research. Thousand Oaks, CA: Sage.

[B54] Stern D. N. (2010). Forms of Vitality: Exploring Dynamic Experience in Psychology, the Arts, Psychotherapy, and Development. Oxford: Oxford University Press.

[B55] Szota K. Schulte K. L. Christiansen H. (2023). Interventions involving caregivers for children and adolescents following traumatic events: a systematic review and meta-analysis. Clin. Child Fam. Psychol. Rev. 26, 17–32. doi: 10.1007/s10567-022-00415-2 36161385 PMC9879828

[B56] Tronick E. (2007). The Neurobehavioral and Social-emotional Development of Infants and Children. New York, NY: WW Norton and Company.

[B57] Tucker C. Schieffer K. Lenz S. Smith S. (2021). Sunshine circles: randomized controlled trial of an attachment-based play group with preschool students who are at-risk. J. Child Adolesc. Couns. 7, 161–175. doi: 10.1080/23727810.2021.1940658

[B58] van der Kolk B. A. (2014). The Body Keeps the Score: Brain, Mind, and Body in the Healing of Trauma. New York, NY: Penguin Books.

[B59] Velu M. E. Kuiper R. M. Schok M. Sleijpen M. de Roos C. Mooren T. (2025). Effectiveness of trauma-focused treatments for refugee children: a systematic review and meta-analyses. Eur. J. Psychotraumatol. 16, 1–20. doi: 10.1080/20008066.2025.2494362 40387621 PMC12090257

[B60] Vulcan M. (2009). Is there any body out there?: a survey of literature on somatic countertransference and its significance for DMT. Arts Psychother. 36, 275–281. doi: 10.1016/j.aip.2009.06.002

[B61] Vulcan M. (2016). “I'm a translating body”: therapists' experiences working with children diagnosed with autism spectrum disorder. J. Psychother. Integr. 26, 326–337. doi: 10.1037/int0000026

[B62] Wang L. Norman I. Edleston V. Oyo C. Leamy M. (2024). The effectiveness and implementation of psychological first aid as a therapeutic intervention after trauma: an integrative review. Trauma Violence Abuse 25, 2638–2656. doi: 10.1177/15248380231221492 38281196 PMC11370167

[B63] Weinberg R. (2025). “Psychoanalytic thoughts on evacuated parents and children during the first weeks of the October 7th war,” in Child and Adolescent Psychoanalysis in Times of Crisis: War, Pandemic, and Climate Change, ed. K. Fiorella (Abingdon: Routledge), 44–65.

[B64] Wisner B. Adams J. (Eds.). (2002). Environmental Health in Emergencies and Disasters: A Practical Guide. Geneva: World Health Organization.

